# Pretreatment setup verification by cone beam CT in stereotactic radiosurgery: phantom study

**DOI:** 10.1120/jacmp.v11i4.3162

**Published:** 2010-08-30

**Authors:** Atsushi Fukuda

**Affiliations:** ^1^ Department of Radiology Shiga Medical Center for Children Moriyama City Shiga Japan

**Keywords:** stereotactic radiosurgery, cone beam CT, setup verification, isocenter difference

## Abstract

Kilovoltage cone beam computed tomography (CBCT) imaging may be useful in verifying patient position in stereotactic radiosurgery (SRS). To evaluate its efficacy, we investigated isocenter differences in the radiation beam and CBCT with respect to the achievable setup of a conventional frame‐based SRS system. A verification phantom constructed from two plastic boards and Gafchromic‐EBT film $\left(4\times 4\;{\text{cm}}^{2}\right)$ pricked with a pin, was scanned by simulation CT. An isocenter at the tip of pin was planned in the treatment planning system and positioned using stereotactic coordinates. Star‐shot irradiation was performed to evaluate the difference between the radiation isocenter and the target (pinhole). CBCT rotation of 200° with a micro multileaf collimator (m3) was performed and measured the isocenter difference between CBCT and the target (tip of pin) by comparing relative coordinates. Data acquisition was performed 13 times on different days and differences were analyzed by calculating mean and standard deviation. The mean difference between the radiation beam and the target (pinhole) and between radiation beam and CBCT isocenter, were 0.6±0.2mm and 0.8±0.1mm, respectively. The setup accuracy of conventional stereotactic coordinates and the isocenter accuracy of CBCT complied with AAPM Report No. 54.

PACS number: 87.53.Ly

## I. INTRODUCTION

Stereotactic radiosurgery (SRS) is a form of radiation therapy for the precise and accurate delivery of radiation to a brain lesion while sparing the surrounding normal tissues. To achieve precise and accurate delivery, it is necessary not only to use special procedures for patient localization such as the use of a stereotactic frame^(^ ^1^ ^)^ but also to perform periodical quality assurance of the linear accelerator (linac) and other apparatus.^(^ ^2^ ^)^ The American Association of Physicists in Medicine (AAPM) published a report on SRS (AAPM Report No. 54)^(^ ^3^ ^)^ that deals with quality assurance criteria and test methods for this procedure, and considers a whole chain of uncertainties. In addition to the above procedure, pretreatment verification of the difference between patient setup and the planned isocenter is crucial for accurate SRS.^(^ ^4^ ^)^

Recently, a new cone beam computed tomography (CBCT) technology has been integrated with the linac and can provide not only high‐resolution, three‐dimensional information about the patient on the treatment couch, but also about the CBCT isocenter position.^(^ ^5^ ^)^ CBCT imaging may therefore be useful in verifying patient position in SRS.^(^ ^6^ ^)^ To evaluate its efficacy, we investigated in the radiation beam and CBCT with respect to the achievable setup accuracy of a conventional frame‐based SRS system.

## II. MATERIALS AND METHODS

### A. Treatment planning

**Figure 1:**
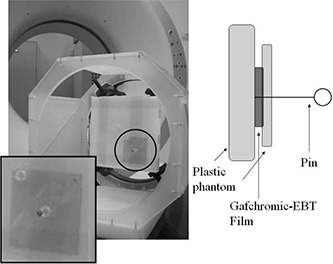
The verification phantom: constructed from two plastic boards, Gafchromic‐EBT film, and a pin; the film with the pin was inserted between the two plastic boards and the tip of the pin was pricked into the film through a small hole in the thinner plastic board.

A verification phantom was constructed from two plastic boards, Gafchromic‐EBT film $\left(4\times 4{\text{cm}}^{2}\right)$ (ISP Corp., Wayne, NJ) and a pin, as shown in Fig. 1. The film was inserted between the two plastic boards. The tip of the pin was pricked into the film through a small hole (1 cm φ) in the thin plastic board. This homemade phantom was attached to a dedicated U‐shaped frame and a CT stereotactic localization box, and was scanned using Sensation Open CT with 20 slices (Siemens AG, Munich, Germany). Images were taken under the following parameters: tube potential 120 kV, 122 mA, quality reference mAs 270, collimation 0.6 mm, rotation time 1.0 s, pitch 0.45, field of view 350 mm, matrix 512×512, and reconstruction at 1.0 mm intervals. CT volume data were transferred to the BrainScan treatment planning system (BrainLAB AG, Feldkirchen, Germany).

The target position relative to the CT stereotactic localization box coordinates was established in the treatment planning system. After the isocenter was planned at the tip of the pin, a target positioner sheet depicting the isocenter was printed out and attached to the target positioner box.

### B. Star shot

**Figure 2:**
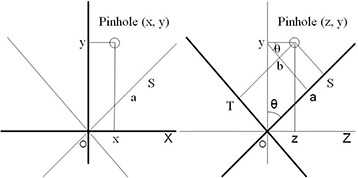
Diagram depicting the irradiated film: (left) the evaluation of the x‐ and y‐coordinates (differences); (right) the evaluation of the z‐coordinate. To calculate the difference in the z‐coordinate, axis rotation was needed. Since Θ was π/4 in this study, z is calculated using Formula (2).

A micro multileaf collimator m3 (BrainLAB AG, Feldkirchen, Germany) was attached to the linac Clinac‐21EX with an On‐Board Imager (Varian Medical Systems, Palo Alto, CA).^(^ ^7^ ^)^ The homemade phantom with the target positioner box was set up using stereotactic coordinates and a room laser. Star‐shot irradiation was performed to evaluate the three‐dimensional differences from a target (a pinhole in the film) to the radiation isocenter, and irradiation was performed with the following parameters: jaw 1×1 cm at couch 0° and 1×9 cm at couch 90°, m3 leaf closed, and 1000 MU per angle. Gantry 0° and 90° (collimator 90°) were adopted for couch rotation 0° to evaluate the lateral (x‐coordinate) and vertical (y‐coordinate) differences. In addition, for the couch rotation 90°, gantry 90° with collimator angle 45° and 135° were adopted to evaluate the longitudinal (z‐coordinate) differences. A diagram depicting the film is shown in Fig. 2. To calculate the difference in the z‐coordinate, axis rotation was adopted according to the following Formulas:

(1) $$\begin{array}{l} S=Oa+aS,\;\;\;T=ay‐yb\\ Oa=y\cos\theta ,\;\;\;aS=z\sin\theta ,\;\;\;ay=y\sin\theta ,\;\;\;\;yb=z\cos\theta \\ S=y\cos\theta +z\sin\theta ,\;\;\;T=y\sin\theta ‐z\cos\theta \end{array}$$

Since Θ is π/4 in this study, these Formulas are converted as follows:

(2) $$z=\left(S\sqrt{2}‐T\sqrt{2}\right)/2$$

The differences (x‐, y‐, z‐coordinates) between the target (pinhole) and the radiation beam were obtained by the use of a 10×magnification loupe.

### C. CBCT

CBCT rotating 200° (gantry 100° to 260°) with m3 was performed at couch rotation 0°. An aluminum bowtie filter was used for all examinations. Images were taken using the following parameters: tube potential 100 kV, tube current 20 mA, slice thickness 1.0 mm, field of view 248 mm, matrix 384×384.

**Figure 3:**
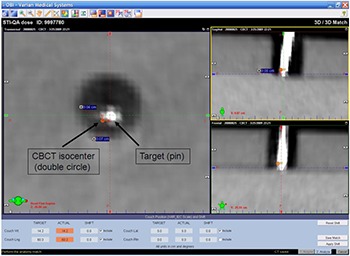
Difference measurement on CBCT workstation. The CBCT isocenter is shown as a double circle in axial, sagittal and coronal images on this workstation. The difference between the CBCT isocenter and the target was obtained using the measurement tool of the CBCT workstation.

The differences (x, y, and z) from the target (tip of pin) to the CBCT isocenter were measured on the CBCT workstation. Furthermore, the correlation of CBCT with the reference dataset was not performed because the simulation CT dataset could not be transferred to the ARIA and OBI workstation (Varian Medical Systems, Palo Alto, CA) through the BrainScan treatment planning system. CBCT axial (blue line), sagittal (red line) and coronal (green line) images were displayed on the CBCT workstation, as shown in Fig. 3. These lines can be set at an arbitrarily position of less than 1 mm CBCT thickness. The position of CBCT images with the tip of the pin (target) was determined to be similar to the simulation CT images with the tip of the pin (planning isocenter). The CBCT isocenter was then superimposed on these views. The difference (distance) from the center of target to the CBCT isocenter was obtained using the measurement tool of the CBCT workstation.

This technique is made feasible by the use of an on‐board imaging system with source axis distance 100 cm and source flat‐panel detector distance 160 cm.

On the other hand, a larger difference (y‐coordinate) between the target and the CBCT isocenter was observed (see below). Relative to the weight of the phantom, the target positioner box may be heavier and cause the fulcrum on the couch mount to flex slightly. To test this hypothesis, similar investigations using CBCT were performed 10 times with and without the target positioner box. Then, the differences (x‐, y‐, and z‐coordinates) of the target (tip of pin) with and without the target positioner box (relative to CBCT isocenter) were measured.

### D. Data assessment

Data acquisition was performed 13 times on different days, and the mean and standard deviation of the x‐, y‐, and z‐coordinate differences and the vector were calculated. Isocenter differences between the radiation beam and CBCT were calculated using relative coordinates. Data are expressed as mean± standard deviation unless otherwise stated.

## III. RESULTS

**Figure 4:**
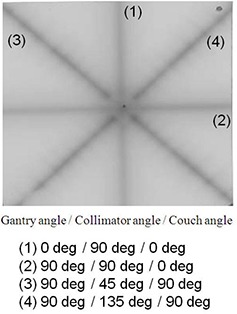
The pinhole (target) and irradiation line were detected clearly to enable measurement of the distances between the target (pinhole) and the radiation isocenter. The differences (x‐, y‐, and z‐coordinates) between the target (pinhole) and the radiation beam were obtained by the use of a 10×magnificationloupe. The film was used for illustration purposes only.

Thirteen star‐shot examinations were successfully performed and one of the irradiated films is shown in Fig. 4. The pinhole (target) and the irradiation line can be detected clearly, and the differences between the target (pinhole) and the radiation isocenter can be measured in all examinations. The differences between the radiation isocenter and the target (pinhole) are shown in Table 1. The differences in the x‐ and y‐coordinates were ‐0.4±0.2mm and ‐0.4±0.2mm, respectively, and the z‐coordinate calculated by Formula (2) was ‐0.1±0.2mm. The vector difference was 0.6±0.2mm (maximum was 0.93 mm).

**Table 1:** Isocenter differences of all data from target to the radiation isocenter measured from the irradiated film (Fig. 4), from the target to CBCT measured on the CBCT workstation, and from the radiation isocenter to CBCT calculated relative difference

*Study Number*	*Target (pinhole) to Radiation Isocenter*			*Target (tip of pin) to CBCT Isocenter*			*Radiation to CBCT Isocenter*		
*Coordinate*	*x*	*y*	*z*	*x*	*y*	*z*	*x*	*y*	*z*
1	‐0.2	0.2	0.4	‐0.3	‐0.6	0.3	‐0.1	‐0.8	‐0.1
2	‐0.4	‐0.5	‐0.4	‐0.5	‐1.3	0.1	‐0.1	‐0.8	0.5
3	‐0.5	‐0.4	‐0.1	‐0.4	‐1.3	0.2	0.1	‐0.9	0.3
4	‐0.4	‐0.5	0.1	‐0.5	‐1.1	0.0	‐0.1	‐0.6	‐0.1
5	‐0.6	‐0.4	‐0.3	‐0.4	‐1.2	‐0.2	0.2	‐0.8	0.1
6	‐0.7	‐0.6	‐0.1	‐0.5	‐1.4	‐0.3	0.2	‐0.8	‐0.2
7	‐0.5	‐0.6	‐0.1	‐0.6	‐1.4	0.0	‐0.1	‐0.8	0.1
8	‐0.3	‐0.1	‐0.1	‐0.4	‐0.8	0.1	‐0.1	‐0.7	0.2
9	‐0.4	‐0.6	‐0.2	‐0.5	‐1.1	0.0	‐0.1	‐0.5	0.2
10	‐0.3	‐0.4	‐0.1	‐0.6	‐1.1	0.0	‐0.3	‐0.7	0.1
11	‐0.1	‐0.3	0.0	‐0.2	‐1.1	0.0	‐0.1	‐0.8	0.0
12	‐0.3	‐0.2	0.0	‐0.5	‐0.9	0.0	‐0.2	‐0.7	0.0
13	‐0.4	‐0.6	‐0.2	‐0.5	‐1.2	‐0.2	‐0.1	‐0.6	0.0
Units are millimeters.									
	*Target (pinhole) to Radiation Isocenter*			*Target (tip of pin) to CBCT Isocenter*			*Radiation to CBCT Isocenter*		
x‐coordinate	‐0.4±0.2mm			‐0.5±0.1mm			‐0.1±0.1mm		
y‐coordinate	‐0.4±0.2mm			‐1.1±0.2mm			‐0.7±0.1mm		
z‐coordinate	‐0.1±0.2mm			0.0±0.2mm			0.1±0.2mm		
Vector	0.6±0.2mm			1.2±0.2mm			0.8±0.1mm		

CBCT examinations were also successfully performed. The tip of the pin (target) could be detected clearly on the CBCT workstation, and the distances between the target (tip of pin) and the CBCT isocenter could be measured in all examinations. The differences between the target (tip of pin) and the CBCT isocenter are shown in Table 1. The differences in x‐, y‐, and z‐coordinates were ‐0.5±0.1mm,‐1.1±0.2mm,0.0±0.2mm, respectively. The vector difference was 1.2±0.2mm (maximum was 1.52).

Isocenter differences between the radiation beam and CBCT were calculated using relative coordinates. The differences between the radiation beam and CBCT are shown in Table 1. The differences in the x‐, y‐, and z‐coordinates were ‐0.1±0.1mm,‐0.7±0.1mm,0.1±0.2mm, respectively. The vector difference was 0.8±0.1mm (maximum was 0.97 mm).

The differences of the target with and without the target positioner box are given in Table 2. The differences in the x‐, y‐, and z‐coordinates were 0.0±0.1mm,‐0.4±0.1mm, and 0.2±0.2mm, respectively (the origin was the target without the target positioner box).

**Table 2:** Differences of targets with and without a target positioner box, measured on the CBCT workstation.

	*Target differences with and without a target positioner box (the origin is without the target positioner box)*
x‐coordinate	0.0±0.1mm
y‐coordinate	‐0.4±0.1mm
z‐coordinate	0.2±0.2mm
Vector	0.5±0.1mm

## IV. DISCUSSION

The American Association of Physicists in Medicine published a report on SRS (AAPM Report No. 54)^(^ ^3^ ^)^ that deals with quality assurance criteria and test methods for this procedure, and considers a whole chain of uncertainties. A criterion of ± 1.0 mm for geometrical accuracy is recommended in this report. The results of the present study show that the accuracy of patient setup using stereotactic coordinates was adequate for SRS (0.6mm<1.0mm), but differences between the target (tip of pin) and the CBCT isocenter exceeded the criteria (1.2mm>1.0mm).

**Figure 5:**
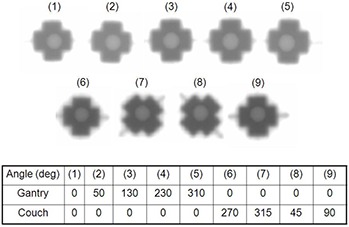
Winston‐Lutz test was used to verify the laser alignment and the stability of gantry and couch rotation. The SRS system in this study was found to be within the criteria of AAPM Report No. 54.

The x‐coordinate difference between the radiation isocenter and the target (pinhole) was ‐0.4±0.2mm. This was comparable to the difference between the CBCT isocenter and the target (tip of pin) (i.e., ‐0.5±0.1mm). This systematic error was investigated using the Winston‐Lutz isocenter test and was found to be within the criteria of AAPM Report No. 54, as shown in Fig. 5.^(^ ^2^ ^,^ ^3^ ^)^ Although laser misalignment was suspected as being a factor for the error, the current laser alignment was valid due to slight gantry instability.

The y‐ coordinate difference between the radiation beam and CBCT was ‐0.7±0.1mm. This difference was relatively large compared to the other differences. CBCT scanning with and without a target positioner box reveals that the target moves up by 0.4±0.1mm (y‐coordinate) due to the weight of the box. Therefore, the differences in the y‐coordinate of the target to the radiation beam and of the target to the CBCT isocenter are 0.0±0.3mm and ‐0.7±0.3mm, respectively, if they are correctly compensated. Yoo et al.^(^ ^5^ ^)^ suggested QA of OBI isocenter accuracy with gantry rotation is required and that the displacement should be less than 2 mm. Furthermore, they showed that maximum vector displacement of about 1.5 mm occurred at a gantry of 180°. It is difficult to correctly compare the vector displacement (1.5 mm) with vector difference (0.8 mm) in this study because Yoo et al. showed isocenter accuracy (two‐dimensions) of OBI and not CBCT. However, the vector difference between the radiation and CBCT isocenter in this study was 0.8±0.1mm. This small difference was not only compatible with their criteria (0.8mm<1.5mm), but was also within the tolerance (0.8mm<1.0mm) shown by the AAPM Report No. 54.^(^ ^3^ ^)^

Chang et al.^(^ ^6^ ^)^ also reported that minor distortion or blurring occurred as a result of the embedded radio‐opaque marker in a RANDO head phantom. This image distortion may seem to produce any significant position error. A pin was used to assess the isocenter difference in the present study. The tip of the pin was very small compared with the radio‐opaque target and did not produce significant image distortion.

There are several limitations of this study. First, these consecutive examinations were performed in a single month. Therefore, long‐term CBCT stability is not clear. Periodical quality assurance for CBCT stability should be considered for safety SRS setup verifications. In our institution, the CBCT system and laser alignment are verified by a monthly quality assurance scheme as proposed by Yoo et al.^(^ ^5^ ^)^ and Rosca et al.,^(^ ^8^ ^)^ respectively.

Also, there is a strict requirement for measurement of the radiation isocenter when performing multi‐angle irradiation because of gantry sag and couch wobble.^(^ ^8^ ^,^ ^9^ ^)^ Although multi‐angle star‐shot irradiation was not performed in this study since the irradiation lines were congested on the film, Formula (1) allows multiple angles. In addition to the verification performed in this study, gantry sag and couch wobble were verified within the 1.0 mm criterion using multi‐angle irradiation in the monthly quality assurance for SRS.^(^ ^3^ ^)^

Another limitation is that the subject was a plastic phantom. If the human brain is scanned by CBCT in pretreatment verification, since the target in the brain may not appear on the CT image without the use of a contrast agent, the distance from the target to the radiation isocenter may not be clear. However, for the evaluation of setup accuracy, bone structure and other anatomical findings will be useful in preventing careless mistakes in patient setup for SRS. Also, it was reported that the repositioning accuracy of mask immobilization was within 2−2.5mm.^(^ ^9^ ^,^ ^10^ ^)^ Willner et al.^(^ ^11^ ^)^ reported the feasibility of simulation CT imaging before SRS treatment to evaluate repositioning accuracy for mask immobilization. If therapists find an unacceptable rotation on the CBCT workstation when the simulation CT data (off‐line in current system) is compared, they should re‐immobilize the patient's head on the treatment couch. Furthermore, therapists can detect human error. Although it is not possible to transfer the simulation CT data set to the CBCT workstation through the BrainScan treatment planning system at our institution, this problem can be overcome by installing a relatively new treatment planning system (iPlan, BrainLAB AG, Feldkirchen, Germany).

Patient setup may become more accurate using CBCT imaging in the near future. Chang et al.^(^ ^6^ ^)^ investigated image fusion, target localization and setup accuracy of CBCT for SRS and reported that the uncertainty of the CBCT setup procedure was on the same order as that of the conventional framed‐based stereotactic systems. However, this technique may require a more accurate positional indicator (potentiometer). The accuracy of commercial couch motors is approximately 1.0 mm or 2.0 mm.^(^ ^5^ ^)^ CBCT will be a useful tool for resetup, using automatic couch shift without reimmobilization on the treatment couch, because the CBCT workstation can be used to calculate the correlation between CBCT and simulation CT on‐line (x‐, y‐, and z‐coordinates, pitch, roll and yaw rotations) using the fusion tool. However, the accuracy of fusion techniques must also be verified prior to clinical use because the error of fusion techniques was not included in the present study. If these limitations are overcome, SRS setup by CBCT imaging and an automatic couch shift system will be feasible in a clinical setting.

Lastly, data acquisitions were performed only 13 times on different days using a single linac with OBI. A large number of test repetitions and verifications of other linac must be performed for the accuracy of the pretreatment QA procedure using CBCT. Therefore, larger studies would be needed to show the efficacy of the pretreatment QA procedure using CBCT in SRS.

## V. CONCLUSIONS

This study was designed to investigate the differences between the (a) radiation isocenter and the target (pinhole), (b) CBCT isocenter and the target (tip of pin), and (c) radiation isocenter and the CBCT isocenter. The results showed that the setup accuracy of conventional stereotactic coordinates and the isocenter accuracy of CBCT complied with AAPM Report No. 54. Therefore, it was found that CBCT imaging before treatment is useful in verifying the setup position and human error. However, the setup using CBCT imaging and an automatic couch shift must be verified prior to clinical use because the accuracy of fusion technique and an automatic couch shift were not included in this study.

## ACKNOWLEDGEMENTS

I am grateful to Shigeki Yamada and Chikako Kawabata of the Department of Radiology, Shiga Medical Center for Adults, for support to this work. I thank Atsuhiko Togashi, Niigata University (retired), for technical discussion.
